# A novel experience-based internet intervention for smoking cessation: feasibility randomised controlled trial

**DOI:** 10.1186/s12889-016-3821-3

**Published:** 2016-11-11

**Authors:** John Powell, Nikki Newhouse, Angela Martin, Sena Jawad, Ly-Mee Yu, Mina Davoudianfar, Louise Locock, Sue Ziebland

**Affiliations:** 1 Health Experiences Research Group, Nuffield Department of Primary Care Health Sciences, University of Oxford, Oxford, UK; 2 Primary Care Clinical Trials Unit, Nuffield Department of Primary Care Health Sciences, University of Oxford, Oxford, UK

**Keywords:** Smoking cessation, Internet intervention, Randomised controlled trial

## Abstract

**Background:**

The internet is frequently used to share experiences of health and illness, but this phenomenon has not been harnessed as an intervention to achieve health behaviour change. The aim of this study was to determine the feasibility of a randomised trial assessing the effects of a novel, experience-based website as a smoking cessation intervention. The secondary aim was to measure the potential impact on smoking behaviour of both the intervention and a comparator website.

**Methods:**

A feasibility randomised controlled single-blind trial assessed a novel, experience-based website containing personal accounts of quitting smoking as a cessation intervention, and a comparator website providing factual information. Feasibility measures including recruitment, and usage of the interventions were recorded, and the following participant-reported outcomes were also measured: Smoking Abstinence Self-Efficacy Questionnaire, the single-item Motivation to Stop Scale, self-reported abstinence, quit attempts and health status outcomes. Eligible smokers from two English regions were entered into the trial and given access to their allocated website for two weeks.

**Results:**

Eighty-seven smokers were randomised, 65 completed follow-up (75 %). Median usage was 15 min for the intervention, and 5 min for the comparator (range 0.5–213 min). Median logins for both sites was 2 (range 1–20). All participant-reported outcomes were similar between groups.

**Conclusions:**

It was technically feasible to deliver a novel intervention harnessing the online sharing of personal experiences as a tool for smoking cessation, but recruitment was slow and actual use was relatively low, with attrition from the trial. Future work needs to maximize engagement and to understand how best to assess the value of such interventions in everyday use, rather than as an isolated ‘dose of information’.

**Trial registration:**

ISRCTN29549695 DOI 10.1186/ISRCTN29549695. Registered 17/05/2013.

## Background

The internet and social media have the potential to deliver accessible, low-cost health behaviour change interventions to smokers [1] who can access them privately and at a time and place of convenience to them. In England almost half of smokers surveyed reported interest in using online smoking cessation interventions [2]. Reviews of internet-based smoking cessation interventions have shown high levels of acceptability and user satisfaction [3], but comparisons of online interventions with usual care have had inconsistent findings [4]. The most promising interventions appear to be those that are structured, include some level of interactivity, and some tailoring to the individual user [3, 4]. More work is needed to determine the most effective elements of internet and social media interventions [1].

One of the key characteristics of current internet use, since the advent of Web 2.0 technologies that have enabled people to share information and interact online, and the widespread uptake of cheap broadband and mobile devices, is the interaction between people in internet settings, and the opportunities for online peer support. Over the last ten years consumers have become “pro-sumers”, producing as well as consuming content, sharing their experiences in blogs, fora, and other forms of social media. Ziebland and Wyke have shown that this internet-based sharing of people’s experience about health issues could influence health through seven domains: finding information, feeling supported, maintaining relationships with others, affecting behaviour, experiencing health services, learning to tell the story, and visualizing disease [5].

Taking the findings of the Ziebland and Wyke [5] conceptual review as the starting point, we attempted to harness the value of other people’s experiences by developing and testing three experienced-based internet interventions (multi-media websites): one for smoking cessation (reported here), and two other interventions which will be reported separately: one for asthma and one for carers of people with multiple sclerosis. This study builds on an emerging evidence base seeking to use digital tools to harness narrative information from peers to influence health behaviour (although not yet in the field of smoking cessation), for example studies on control of hypertension [6], attitudes to breast screening among African American women [7, 8], and lifestyle change for people with coronary heart disease and low-back pain [9].

For smokers, social support can be of benefit in quitting [10], although the evidence base is relatively limited [11, 12]. Peer support interventions may be of particular value to smokers who have fewer opportunities for informal social support [13]. A study of pregnant smokers showed that they valued high levels of personalized online support (from peers and ‘experts’) while quitting, maintaining abstinence and managing relapse [14], and there is an emerging literature on the value of online social networking in smoking cessation [15]. As described in the Theory of Planned Behaviour, social norms have an influence in health behavior change, as has been well recognized in the field of smoking cessation (e.g. [16]). Online social contact may also help smokers who are contemplating cessation, by both providing new ideas about strategies to try, and by helping to give the strategies authenticity, salience and meaning. It may have particular authority for those who feel that non-smoking health professionals (and family, friends, or colleagues) are not able to empathise with the challenges of quitting.

Brandt and colleagues [17] found that the majority of messages on a blog associated with a smoking cessation intervention could be categorized as ‘personal stories and experiences’ (53 %) or ‘emotional support’ (34 %) and that members of the blog could be inspired and motivated [17]. These authors propose social comparison as a mechanism for the beneficial effect of these messages: that users wanting to quit are motivated by stories from successful quitters; and that successful quitters are motivated to remain abstinent by reading posts from those who are in the early stages of quitting. This is supported by previous work on using testimonials to support smoking cessation which has shown that both inspiring ‘how to quit’ positive personal stories, as well as more negative accounts conveying ‘why to quit’ messages, can encourage quitting [18].

The primary aim of this study was therefore to determine the feasibility of trialing this novel experience-based website containing personal accounts of quitting as a smoking cessation intervention. The secondary aim was to report the effect of the intervention and comparator websites on various self-reported outcome measures including motivation to stop smoking, abstinence rates, and smoking abstinence self-efficacy.

## Methods

### Participants

Current smokers who indicated some willingness to quit were recruited through sixteen primary care practices in two regions of England between June 2013 and August 2014. Practices were identified and reimbursed through the local primary care research network and initiated as recruitment sites by the trial manager. Individuals were eligible if they had been smokers for at least one year, had some interest in quitting, were aged 18 years or over, lived in England and had internet access. We recruited men and women. Individuals were excluded if they were unable to read English or if they were terminally ill or had another significant disease or disorder which, in the opinion of the GP, may either put that person at risk because of participation in the study, or may influence the result of the study, or affect that person’s ability to participate in the study. General practitioners were asked to identify potentially eligible individuals and to mail out study participation invitation letters and information sheets with details of how to take part. Potential participants were required to contact the study team who then sent a consent form, and once this was returned, participants were invited to complete an eligibility screening questionnaire and enrolled.

### Interventions

The ‘experience-based’ intervention was a multi-media website developed by the University of Oxford Health Experiences Research Group and the Healthtalk.org charity, guided by an advisory panel that included leading clinicians, researchers, and lay representatives. The content included text, video and audio extracts from qualitative interviews with 34 ex-smokers, who had been purposively recruited to capture a diverse range of experiences. The interviews were analysed qualitatively using a thematic approach to identify the common, important themes [19]. As a result, the research team prepared analytic summaries of 22 separate topics with over 250 illustrative video and audio extracts chosen from across the set of the interviews. These topic summaries with illustrative personal accounts were then presented on the website as a menu of topics which a user could browse and navigate in whichever way they chose. The website was not personalized to the individual user: all users experienced the same website and they could browse it in any order they chose. There was no ‘tunneling’ and no interactivity other than the option to provide feedback to the website team. Example topics were: first thinking about quitting; life events and their effect on people’s motivation to stop smoking; the role of others in the decision to quit; cutting down, unsuccessful attempts and trying again. The selection of topics, the wording of the summaries and the selection of illustrative clips were all guided by the advisory panel to help ensure that the site encapsulated the value of hearing a wide range of other people’s experiences of giving up smoking.

In this feasibility study we wanted to compare against a high quality information-based alternative. We therefore provided a website based on facts and figures about smoking cessation taken from NHS Choices (www.nhs.uk) the leading authoritative provider of online health information in the UK. This comparator site contained no personal experience information. We constructed a new site which had the look and feel of our intervention site (same design features, including video and audio clips of health professionals talking about smoking cessation, same colours and logos, same navigation, etc.) and contained ‘facts and figures’ information.

### Design and procedures

This was a randomised, controlled, single-blind study. Potential participants completed an eligibility screening questionnaire and provided consent. They then completed baseline measures (using an online portal), after which they were randomised to have access to either the intervention or comparator website. Randomisation used a computer-generated random number sequence in a 1:1 ratio, created by an independent statistician. Each participant created their own username and password allowing unlimited access to their allocated website for a two-week period. After this participants were invited (by email) to complete final follow-up measures. Up to two emails and one telephone call were used as reminders. Participants could not be blind to the nature of the website to which they were allocated but they did not know whether they were receiving the comparator or the intervention website, only that we were evaluating two approaches to giving health information. Those conducting the analysis were blinded to allocation. We also conducted interviews with the participants in this trial and with participants in the two other trials attempting to harness online personal experiences as interventions and the joint analysis of these (across all 3 studies) will be reported separately [20].

### Outcome measures

As a feasibility study, our primary measures concerned the number of participants consented and recruited, use of the websites (numbers of logins, page views, and time on site) and the numbers with completed outcome measures or lost to follow-up. We also used various self-report measures at two weeks post-randomisation to the website, mainly to test the feasibility of using these measures as for many of these we would not expect significant change in a short period of time: Motivation to Stop Scale (MTSS, single item with an 8-point scale); abstinence rates (single question); quit attempts (single question); Smoking Abstinence Self-efficacy Questionnaire (SASEQ, 6-item); and the 36-item Short Form Health Survey (SF36) (including both physical and mental dimensions and all subscales). Finally, we used the E-Health Impact Questionnaire (eHIQ) which captures the attitudes of respondents towards the website they have recently viewed [21, 22]. The eHIQ consists of two independently administered and scored parts (eHIQ-Part 1 and eHIQ-Part 2). The eHIQ part 1 consists of 11 items asking about a person’s general attitudes towards health-related websites. The eHIQ part 2 consists of 26 items asking about a person’s views regarding a specific health-related website. We therefore only used part-2 as an outcome measure.

### Sample size and statistical analyses

As described above, this smoking cessation study was one of three feasibility studies harnessing patient experiences as interventions and the target sample size for each study was 100 participants, with the feasibility the primary determinant of this. In terms of effect sizes, based on a balanced 1:1 randomisation, the study would detect potential large effects of the intervention: for dichotomous outcomes these were equivalent to relative risks of 2.1 or above for a baseline rate of 30 % or less given an alpha of 0.05 and 90 % power; for continuous outcomes these detectable differences would be of the order of 0.4 standard deviations (SD) based on the same power and significance. Feasibility outcome measures were summarized using descriptive statistics such as rates reported as percentages. Formal hypothesis testing was not performed in this feasibility study. However, estimated difference in outcome measures of efficacy and corresponding 95 % confidence intervals were calculated using analysis of covariance (ANCOVA) adjusting for baseline values.

## Results

**Fig. 1:**
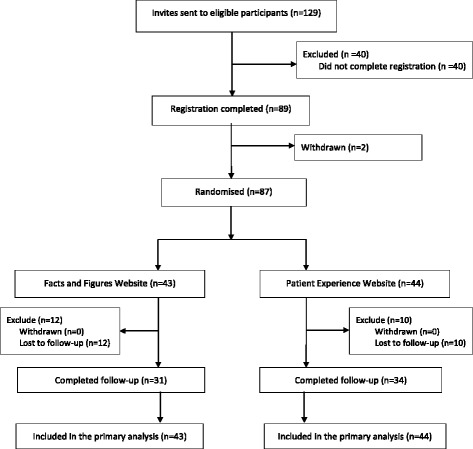
CONSORT flow diagram

129 invitations were sent in response to expressions of interest in the study, and 89 individuals (69 %) completed registration (i.e. consented and completed baseline questionnaires). Two participants withdrew before randomisation, leaving 87 randomised participants. 44 of these (51 %) were allocated to the patient experience website. Of the randomised participants 22/87 (25 %) were lost to follow-up. Figure 1 shows the CONSORT flow diagram.

Table 1 shows baseline demographic and smoking characteristics of participants. Forty-five of the 87 participants (52 %) were women. Most participants were regular internet users who rated their internet ability as ‘good’ to ‘excellent’. Most reported smoking between 11 and 20 cigarettes per day (53 % of the sample). Although most had intentions to quit smoking, despite our inclusion criteria, 19 participants (22 %) reported either not wanting to quit (3 %, *n* = 3) or knowing that they should but not ‘really’ wanting to (18 %, *n* = 16). 27 participants reported having made an attempt to quit during the 2 weeks before starting the study (31 %).

**Table 1:** Baseline demographic and smoking characteristics of the study population

	Comparator (*n* = 43)		Intervention (*n* = 44)	
	*N*	(%)	*N*	(%)
Age				
Mean (SD) {Min-Max}	53.6 (15.1) {17–80}		55.1 (12.9) {24–92}	
Gender				
Female	23	(53.5)	22	(50.0)
Male	20	(46.5)	22	(50.0)
Self-rated ability to use the internet				
Excellent	9	(20.9)	10	(22.7)
Good	15	(34.9)	21	(47.7)
Fair	16	(37.2)	11	(25.0)
Poor	3	(7.0)	2	(4.5)
Bad	0	(0)	0	(0)
Internet use				
At least once a day	26	(60.5)	28	(63.6)
Several times a week	17	(39.5)	10	(22.7)
Once a week	0	0	3	(6.8)
Less than once a week	0	0	3	(6.8)
Ethnicity				
White- English/ Welsh/ Scottish/ Northern Irish/ British	39	(90.7)	41	(93.2)
White- Any other White background	2	(4.7)	2	(4.5)
Mixed	1	(2.3)	0	0
Asian - Pakistani	0	0	1	(2.3)
Black - Caribbean	1	(2.3)	0	0
Number of cigarettes smoked per day				
10 or less	12	(27.9)	15	(34.1)
11–20	25	(58.1)	21	(47.7)
21–30	4	(9.3)	7	(15.9)
31 or more	2	(4.7)	0	0
Did not answer	0	0	1	(2.3)
Time from wake up to smoke first cigarette				
After 60 min	8	(18.6)	13	(29.5)
31–60 min	9	(20.9)	11	(25.0)
6–30 min	17	(39.5)	15	(34.1)
Within 5 min	8	(18.6)	4	(9.1)
Did not answer	1	(2.3)	1	(2.3)
Serious attempt to stop smoking in past 2 weeks				
No	32	(74.4)	27	(61.4)
Yes	11	(25.6)	16	(36.4)
Did not answer	0	0	1	(2.3)
Which of the following describes you?				
I don’t want to stop smoking	1	(2.3)	2	(4.5)
I think I should stop smoking but don’t really want to	9	(20.9)	7	(15.9)
I want to stop smoking but haven’t thought about when	5	(11.6)	2	(4.5)
I REALLY want to stop smoking but I don’t know when I will	15	(34.9)	16	(36.4)
I want to stop smoking and hope to soon	6	(14.0)	6	(13.6)
I REALLY want to stop smoking and intend to in the next 3 months	2	(4.7)	4	(9.1)
I REALLY want to stop smoking and intend to in the next month	3	(7.0)	5	(11.4)
Don’t know	2	(4.6)	2	(4.6)

The recorded usage of the websites is shown in Table 2. This does not include time spent on study enrolment or completing measures. This shows that the median number of logins in the two-week period for both sites was 2, with a median number of page views of 12 for the intervention site and 7 for the comparator site. The median duration of use of the patient experience site was 15 min, and for the comparator site was 5 min. According to these data, one user never logged in to their allocated site, and two other users logged in but did not record a page view or spend any time on the site. The accuracy of this data is discussed in the limitations section below.

**Table 2:** Usage data for comparator and intervention websites

	All participants (*n* = 87)	Comparator (*n* = 43)	Intervention (*n* = 44)
Total number of logins to website			
*N*	86	42	44
Median (range)	2 (1 to 20)	2 (1 to 8)	2 (1 to 20)
{Interquartile range}	{1 to 3}	{1 to 3}	{1.5 to 3}
Total number of pages visited			
*N*	84	42	42
Median (range)	10 (1 to 237)	7 (1 to 225)	11.5 (1 to 237)
{Interquartile range}	{4 to 24.5}	{3 to 20}	{5 to 30}
Total duration on site (minutes)^a^			
*N*	86	42	44
Median (range)	9 (0.5 to 213)	5 (0.5 to 69)	15 (0.5 to 213)
{Interquartile range}	{1 to 26}	{1 to16}	{3 to 35}

^a^Units of total duration was recorded in mins, for participants with 0 mins recorded we approximated this to 0.5 mins

No adverse events or harms were reported. The mean Smoking Abstinence Self-Efficacy Questionnaire (SASEQ) scores were similar between the randomised groups. The baseline mean score (SD) for the intervention group was 1.94 (1.02) and for the comparator group 1.84 (0.89), at 2-week follow-up these scores were 1.89 (0.99) and 1.71 (0.95) respectively. Both groups had low mean SASEQ scores indicating low self-confidence in abstaining from smoking in a variety of situations. An ANCOVA found an adjusted mean difference for the change in SASEQ scores between the two randomised groups of 0.155 (95 % CI −0.262 to 0.573), adjusted for baseline SASEQ scores (i.e. showing no significant difference).

The findings from the other exploratory comparisons of the smoking-specific outcome measures are shown in Table 3. This table shows that the baseline and follow-up scores and the change scores for the Motivation to Stop Scale, abstinence rates, and quit attempts, were similar between groups for baseline and follow-up measures and for change scores.

**Table 3:** Exploratory comparisons between randomised groups on smoking specific measures

	All participants (*n* = 87)		Comparator (*n* = 43)		Intervention (*n* = 44)	
	*N*	(%)	*N*	(%)	*N*	(%)
Motivation to Stop Scale (MTSS)						
Change in MTSS from baseline						
Decreased Motivation	14	(16)	6	(14)	8	(18)
Improved Motivation	19	(22)	9	(20)	10	(23)
No Change	28	(32)	14	(33)	14	(32)
Unknown	26	(30)	14	(33)	12	(27)
Abstinence at follow-up						
Cigarettes or other tobacco used in the last 7 days at 2 weeks						
Yes	57	(65)	28	(65)	29	(66)
No	8	(9)	3	(7)	5	(11)
Unknown	22	(25)	12	(28)	10	(23)
Quit attempts						
Quit attempts in the last 2 weeks at baseline						
Yes	27	(31)	11	(26)	16	(36)
No	59	(68)	32	(74)	27	(62)
Unknown	1	(1)	0	0	1	(2)
Quit attempts in the last 2 weeks at 2 weeks						
Yes	25	(29)	11	(26)	14	(32)
No	39	(45)	20	(46)	19	(43)
Unknown (loss to follow up)	23	(26)	12	(28)	11	(25)

Table 4 shows the results for the E-Health Impact Questionnaire part 2 indicating very slight differences between groups, favouring the comparator “facts and figures” site, both in the overall score and in the “information and presentation” and “understanding and motivation” subscales (although in all cases the upper 95 % confidence interval limit was very close to no difference).

**Table 4:** Mean (SD) of eHIQ part 2 scores for intervention and comparator group at two-week follow-up

	Comparator (*n* = 30/43)	Intervention (*n* = 31/44)	Unadjusted mean difference	95 % CI
eHIQ part 2 overall scores	68.71 (0.46)	62.99 (0.38)	−5.718	−10.928 to −0.509
Subscales				
Confidence and Identification	61.74 (12.06)	59.20 (10.11)	−2.537	−8.137 to 3.063
Information and Presentation	73.79 (10.85)	68.37 (10.71)	−5.419	−10.806 to −0.033
Understanding and Motivation	69.63 (14.20)	61.72 (12.91)	−7.911	−14.798 to −1.024

The results for the SF-36 were similar between groups. Participants in both arms showed non-significant improvement in the mental component and a very small, non-significant worsening in the physical component (see Table 5). There was no visible trend between the two groups. The adjusted mean differences between the two groups (ANCOVA analysis, *n* = 64 participants with full data, data not shown in tables) for the absolute change in physical component score of the SF-36 was 1.393 (95 % CI −0.702 to 3.489) and for the absolute change in mental component score was 0.785 (95 % CI −3.09 to 4.648) (no significant differences between groups).

**Table 5:** Summary statistics for the SF36 health status physical component (PCS) and mental component (MCS) summary scores by randomised group

	Comparator (*n* = 43)			Intervention (*n* = 44)		
	*N*	Mean (SD)	{Min, Max}	*N*	Mean (SD)	{Min, Max}
Baseline PCS	43	44.08 (11.02)	{19.63, 64.91}	44	48.59 (8.41)	{24.91, 61.07}
2-week PCS	31	43.21 (11.10)	{19.85, 61.88}	33	48.43 (8.76)	{21.29, 63.73}
Change in PCS from baseline	31	−0.92 (4.12)	{−11.74, 5.92}	33	−0.14 (4.49)	{−7.77, 14.20}
Baseline MCS	43	43.00 (13.57)	{10.56, 65.69}	44	46.68 (11.19)	{17.94, 67.15}
2-week MCS	31	43.42 (15.18)	{10.45, 69.15}	33	47.99 (10.25)	{25.38, 62.54}
Change in MCS from baseline	31	2.20 (8.79)	{−19.14, 26.82}	33	1.89 (7.44)	{−10.78, 21.02}

## Discussions

The primary objective of this trial was to assess the feasibility of using a randomised trial to measure the impact of a novel internet intervention which attempts to harness patient experiences for behaviour change. The intervention was delivered to participants and there were no adverse events or protocol deviations. Our findings show that to a certain extent it was feasible to use a primary care approach to recruit and consent people who smoke to the study, although recruitment took a long time, and we only recruited 87 % of our target sample size (87/100). There has only been limited previous research into the optimum recruitment of participants to internet-based smoking cessation interventions, with a recent study suggesting the benefit of a combination of offline and online routes [23], and other studies have shown that online only approaches can yield high recruitment [24]. Our questionnaire responses suggest that the eligibility criteria were not always applied correctly: some smokers had already given up, while others had little desire to quit. It may be difficult to identify, through primary care, people who are poised to give up smoking yet are not currently trying, and who are willing to take part in a trial. We relied on primary care practices identifying potential participants according to the eligibility criteria, and they were free to do so in whichever way they chose. This limitation may have introduced some bias in the recruited population. Also, as might be expected for this type of intervention, the recruited participants had a relatively high self-rated ability to use the internet. By chance the intervention group contained more participants who used the internet once a week or less (*n* = 6) than the comparator group (*n* = 0).

This study was not designed to assess efficacy, but our comparisons between randomised groups showed no important differences on the self-report measures that included smoking-specific outcomes and health status. The eHIQ findings suggested that the participants rated the intervention site slightly lower for items reflecting trust and suitability of the content, and items reflecting learning relevant information and motivation to take action. In this study the comparator group received access to ‘gold standard’ factual information about smoking cessation, so it would be surprising if our novel intervention containing personal accounts of stopping smoking showed a major comparative benefit. The experience-based intervention group showed no harm compared with the fact-based website comparator.

Retention rates (75 % retention at two-weeks) were moderate, considering the short period of follow-up. We sent up to two reminder emails to participants to complete their self-report follow-up measures, and one telephone contact. Other work has shown the high levels of attrition in online trials of fully automated internet interventions, and the value of more intensive contact in reducing this [25, 26]. In future work we would recommend more telephone or SMS contact in addition to email to help minimize attrition. The absence of face-to-face contact is one attraction of internet trials for public health interventions as it lowers cost and allows them to be more geographically dispersed, but there is a trade-off between this lack of personal contact and rates of loss to follow-up.

We used a period of two weeks during which we invited participants to access their allocated website. In practice the median number of recorded logins was 2, and the median amount of time spent on the site was 10 min, although there was wide variation (up to 4 h ‘exposure’). This low actual use of an internet intervention is common in many e-health trials [27], and the plausibility of this level of exposure having an effect must be questioned. Future work should seek to maximize engagement with the intervention. McClure et al. [28] showed how intervention design can influence engagement with online smoking cessation, with relatively ‘directive’ design measures including a prescriptive message tone, dictating content viewing order, and sending reminder emails, all increasing engagement [28].

Future work should also address the issue of what constitutes a ‘dose’ of online information. We mean this in both a ‘dose as concept’ sense (should an information-based intervention be conceptualised as a ‘dose’ and assessed in the same ways as medicines), as well as in a ‘dose as measurement’ sense (how much of the intervention is required to have an effect). In a real world setting experiential information is rarely isolated from other resources, and internet users do not all use websites in the same way or ‘consume’ them in a two-week period. Internet users search and browse, and do not generally visit ‘on prescription’. Understanding and interpreting the level of use and the nature of engagement with a digital intervention in a trial setting, and drawing conclusions relevant to everyday use, represent major current challenges in digital heath research.

This was feasibility work and the next steps for this research, before progressing to a larger efficacy study with adequate power and length of follow-up, are as follows. Firstly, to refine the intervention, ensuring any further development is grounded in health psychology theory of behaviour change (for example using the Theory of Planned Behaviour, given the potential influence of the intervention on normative beliefs and subjective norms). Secondly, to ensure future recruitment more accurately and rapidly identifies eligible participants. Thirdly to maximize engagement with both the intervention and the trial, for example by using reminders and considering a more pragmatic design where use of the intervention fits more into everyday internet use, alongside other sources of help and information. In this work we compared against a high quality information alternative, as we wanted to determine whether any possible harms might arise with the novel intervention, compared with the information usually provided by the NHS. Future evaluation should determine the value of this intervention in addition to other smoking cessation approaches. It should also identify whether there are certain user groups that this type of intervention may benefit - perhaps groups who have low levels of social support, or perhaps people who have already quit and need help to maintain abstinence (in a qualitative study of online forum posts, Burri et al. analyzed the forum aimed at recent ex-smokers because it was far more active than forums aimed at current smokers [29]). Future work could also assess the potential value in being able to *tell* your story, as well as the opportunity for an experience-based intervention to be changed by its users, by examining interactive approaches to peer support. In this present study we used a curated website containing personal accounts which had been solicited and rigorously analysed, and which could be presented to each participant in the same way. In practice people who are giving up smoking may choose to contribute their own experiences and comments, as well as look at others, and websites which gather experiences tend to evolve over time. Future studies should also consider that barriers and facilitators to the wider implementation of this novel approach.

## Conclusions

Smoking remains a major challenge for global public health. Alongside successful legislative measures such as changes to taxation or advertising rules, there remains a need for innovative approaches and resources to support or motivate smokers to give up [1]. The internet is playing a significant role in health and health care and offers one opportunity for innovative, low cost, highly scaleable approaches to smoking cessation. Harnessing online personal experiences of health and illness to deliver public health benefit is a new approach and we have shown this can be packaged as an intervention and we have tested the feasibility of assessing its impact in a randomised controlled trial. Future work needs to not only address the feasibility issues and investigate efficacy, but also determine the place of such an intervention alongside other tools, as well as methods to maximise engagement. In the real world, those smokers who are interested in seeking information or support online will increasingly find their own way to the websites that appeal to them when they are ready, which may be hard to predict. People considering a behaviour change such as smoking cessation are unlikely to use a single website at a single time, and future work should allow for more naturalistic access and use of such interventions.

## Abbreviations

ANCOVA: Analysis of co-variance
CONSORT: Consolidated standards of reporting trials
eHIQ: E-Health Impact Questionnaire
MTSS: Motivation to Stop Scale
NHS: National Health Service
SASEQ: Smoking Abstinence Self-efficacy Questionnaire
SD: Standard deviation
SF36: Short form health survey
UK: United Kingdom

## References

[1] Borrelli B (2010). Smoking cessation: next steps for special populations research and innovative treatments. J Consult Clin Psychol.

[2] Brown J, Michie S, Raupach T (2013). Prevalence and characteristics of smokers interested in internet-based smoking cessation interventions: cross-sectional findings from a national household survey. J Med Internet Res.

[3] Shahab L, McEwen A (2009). Online support for smoking cessation: a systematic review of the literature. Addiction.

[4] Civljak M, Stead L, Hartmann-Boyce J (2013). Internet-based interventions for smoking cessation. Cochrane Database Syst Rev.

[5] Ziebland S, Wyke S (2012). Health and illness in a connected world: How might sharing experiences on the internet affect People’s health?. Milbank Q.

[6] Ashton CM, Houston TK, Williams JH, Larkin D, Trobaugh J, Crenshaw K (2010). A stories-based interactive DVD intended to help people with hypertension achieve blood pressure control through improved communication with their doctors. Patient Educ Couns.

[7] Perez M, Sefko JA, Ksiazek D, Golla B, Casey C, Margenthaler JA (2014). A novel intervention using interactive technology and personal narratives to reduce cancer disparities: African American breast cancer survivor stories. J Cancer Surviv.

[8] Kreuter MW, Holmes K, Alcaraz K, Kalesan B, Rath S, Richert M (2010). Comparing narrative and informational videos to increase mammography in low-income African American women. Patient Educ Couns.

[9] Schweier R, Romppel M, Richter C, Hoberg E, Hahmann H, Scherwinski I (2014). A web-based peer-modeling intervention aimed at lifestyle changes in patients with coronary heart disease and chronic back pain: sequential controlled trial. J Med Internet Res.

[10] Brothers B, Borrelli B (2011). Motivating Latino smokers to quit: does type of social support matter?. Am J Health Promot.

[11] May S, West R (2000). Do social support interventions (“buddy systems”) aid smoking cessation? A review. Tob Control.

[12] Webel A, Okonsky J, Trompeta J (2010). A systematic review of the effectiveness of peer-based interventions on health-related behaviors in adults. Am J Public Health.

[13] Ford P, Clifford A, Gussy K (2013). A systematic review of peer-support programs for smoking cessation in disadvantaged groups. Int J Environ Res Public Health.

[14] Herbec A, Beard E, Brown J (2014). The needs and preferences of pregnant smokers regarding tailored Internet-based smoking cessation interventions: a qualitative interview study. BMC Public Health.

[15] Cheung YTD, Chan CHH, Lai C-KJ (2015). Using WhatsApp and facebook online social groups for smoking relapse prevention for recent quitters: a pilot pragmatic cluster randomised controlled trial. J Med Internet Res.

[16] Wiltshire S, Bancroft A, Parry O (2003). ‘I came back here and started smoking again’: perceptions and experiences of quitting among disadvantaged smokers. Health Educ Res.

[17] Brandt C, Dalum P, Skov-Ettrup L (2013). ‘After all - It doesn’t kill you to quit smoking’: an explorative analysis of the blog in a smoking cessation intervention. Scand J Public Health.

[18] Durkin S, Brennan E, Wakefield M (2012). Mass media campaigns to promote smoking cessation among adults: an integrative review. Tob Control.

[19] Ziebland S, McPherson A (2006). Making sense of qualitative data analysis: an introduction with illustrations from DIPEx (personal experiences of health and illness). Med Educ.

[20] Ziebland S, Powell J, Briggs P, Jenkinson C, Wyke S, Sillence E, et al. Examining the role of patients’ experiences as a resource for choice and decision-making in healthcare: a creative, inter-disciplinary mixed method study in digital health. Programme Grants Appl Res 2016 (in press). 27929620

[21] Kelly L, Jenkinson C, Ziebland S (2013). Measuring the effects of online health information for patients: item generation for an e-health impact questionnaire. Patient Educ Couns.

[22] Kelly L, Ziebland S, Jenkinson C (2015). Measuring the effects of online health information: scale validation for the e-health impact questionnaire. Patient Educ Couns.

[23] Heffner JL, Wyszynski CM, Comstock B, Mercer LD, Bricker J (2013). Overcoming recruitment challenges of web-based interventions for tobacco use: the case of web-based acceptance and commitment therapy for smoking cessation. Addict Behav.

[24] Brown J, Michie S, Geraghty AWA, Yardley L, Gardner B, Shahab L (2014). Internet-based intervention for smoking cessation (StopAdvisor) in people with low and high socioeconomic status: a randomised controlled trial. Lancet Respir Med.

[25] Wangberg SC, Bergmo TS, Johnsen JA (2008). Adherence in internet-based interventions. Patient Prefer Adherence.

[26] Geraghty A, Torres L, Leykin Y (2012). Understanding attrition from international internet health interventions: a step towards global eHealth. Health Promot Int.

[27] Kohl LF, Crutzen R, de Vries NK. Online Prevention Aimed at Lifestyle Behaviors: A Systematic Review of Reviews. Eysenbach G, ed. Journal of Medical Internet Research. 2013;15(7):e146. doi:10.2196/jmir.2665 10.2196/jmir.2665 PMC3714003 23859884

[28] McClure JB, Shortreed SM, Bogart A, Derry H, Riggs K, St John J, Nair V, An L (2013). The effect of program design on engagement with an internet-based smoking intervention: randomized factorial trial. J Med Internet Res.

[29] Burri M, Baujard V, Etter J (2006). A qualitative analysis of an Internet discussion forum for recent ex-smokers. Nicotine Tob Res.

